# Treg Control of CD80/CD86 Expression Mediates Immune System Homeostasis

**DOI:** 10.1002/eji.202551771

**Published:** 2025-05-09

**Authors:** Yong‐Hee Kim, Abir K. Panda, Ethan M. Shevach

**Affiliations:** ^1^ Cellular Immunology Section Laboratory of Immune System Biology National Institute of Allergy and Infectious Diseases National Institutes of Health Bethesda Maryland USA

**Keywords:** CLTA‐4, costimulation, Foxp3, interferon‐gamma, scurfy mouse

## Abstract

Foxp3^+^ regulatory T cells (Treg) are critical for the maintenance of self‐tolerance, and their absence or dysfunction can result in autoimmunity. To determine the critical cell type controlled by Treg and potentially the suppressor mechanism utilized by Treg in the steady state, we utilized mice expressing the diphtheria toxin receptor (DTR) exclusively on Treg cells. Complete depletion of Treg was achieved 24 h after DT treatment, but profound activation of CD4^+^ and CD8^+^ T cells as measured by induction of CD44 expression and proliferation required 3–4 days. Increased expression of CD80/CD86 was observed on dendritic cells and more prominently on macrophages after 3 days. Depletion of CD4^+^ T cells or macrophages resulted in ∼50% inhibition of T‐cell activation. The initial steps in T‐cell activation were completely independent of IFN‐γ or IL‐2, while upregulation of CD80/CD86 was partially dependent on IFN‐γ. Complete reversal of immune activation post‐Treg depletion was only achieved by blockade of CD80/CD86 interactions with CD28. We conclude that the major mechanism used by Treg in the steady state is the regulation of CD80/CD86 expression and dysregulation of this suppressor pathway results in lethal autoimmunity driven by co‐stimulatory signals in concert with TCR stimulation, or even by costimulatory signals alone.

## Graphical abstract

The acute depletion of Tregs induces activation of APCs and Tconvs, eventually leading to autoimmune disease. This activation could only be prevented by the blockade of CD80/CD86, so CD80/CD86‐CTLA‐4 interactions appear to be the most important Treg suppressor mechanism in the maintenance of immune homeostasis in the steady state.

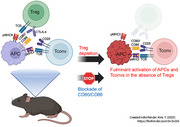

**Abbreviations**
DT: diphtheria toxin
DTR: diphtheria toxin receptor
RT: room temperature
Tconv: conventional T cell
Treg: regualtory T cell

## 1 Introduction

Regulatory T (Treg) cells expressing the lineage‐defining transcription factor, Foxp3 [1, 2, 3], play an essential role in self‐tolerance [4]. Not surprisingly, mutations in the *FOXP3* gene can cause a fatal multiorgan autoimmunity called immune dysregulation, polyendocrinopathy, enteropathy, and X‐linked (IPEX) syndrome in humans, and its genetic equivalent, the scurfy mouse [5]. While mutations in the *FOXP3* gene result in the development of autoimmunity at birth, the seminal studies by the Rudensky lab and others [6, 7, 8] demonstrating that depletion of Foxp3 expressing Treg from adult transgenic mice expressing the diphtheria toxin receptor (DTR) under the control of the *Foxp3* promoter (Foxp3‐DTR) also results in the rapid development of a multiorgan inflammatory syndrome establish that Treg plays a critical role in the maintenance of immune homeostasis in the steady state. Furthermore, the demonstration that commensal microbiota is not essential for the induction of autoimmunity post‐Treg depletion [9] demonstrates that Tregs are controlling responses to self or potentially environmental antigens. These results strongly imply that Tregs are actively exerting their immune suppressive function(s) continually in the steady state, but the mechanism(s) used by Treg remain poorly characterized.

It is widely accepted that Treg cells can utilize a spectrum of suppressor mechanisms and the selection of a given mechanism is likely to be context‐dependent [10, 11]. For example, the suppression of inflammatory disease of the colon requires the production of IL‐10 by Treg, while the milder inflammatory milieu of autoimmune gastritis can be suppressed by Treg in an IL‐10‐independent manner [12]. In this study, we utilize the Foxp3‐DTR mouse model to examine the early events that occur after Treg depletion. Our overall aim is to identify the initial cell type targeted and potentially the suppressor mechanism utilized by Treg in the steady state. The approach we have used is to simultaneously deplete Treg and a given T cell or APC population and quantitate the effects of this procedure on early events in T‐cell activation, as measured by upregulation of CD44 expression and Ki‐67 expression as well as cytokine production and expression of costimulatory molecules on different APC populations. While both T cells and APC play critical roles in the early events of immune activation seen after Treg depletion, the major initiating event appears to be the dysregulation of Treg control of APC‐mediated co‐stimulation as reflected by our ability to completely block all aspects of immune activation seen after Treg depletion by blocking CD80/CD86. The implications of this result via defects in Treg function in autoimmunity and manipulation of immune function by CTLA‐4‐Ig therapy will be discussed.

## 2 Results

### 2.1 Conventional T Cells (Tconv) Are Rapidly Activated After Treg Depletion

**FIGURE 1:**
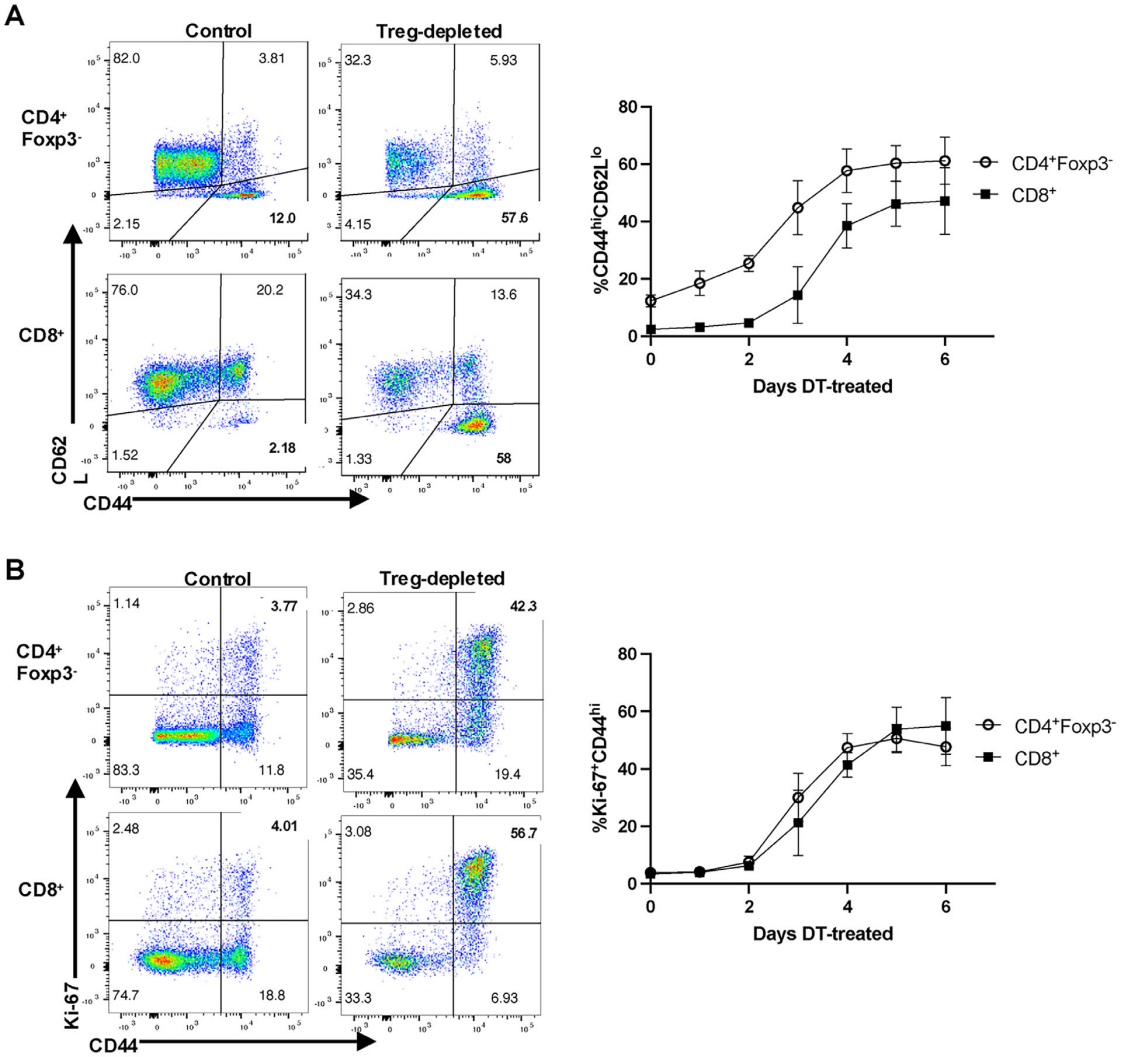
Rapid activation of Tconv cells after Treg depletion. Splenocytes were analyzed after the Treg depletion with diphtheria toxin (DT). Control mice were given PBS (InVivoPure pH7.0 Dilution Buffer). Representative 2D plots are 6 days after the initiation of DT treatment. Cells were gated on live TCRβ^+^CD4^+^Foxp3^‐^GFP^‐^ cells or TCRβ^+^CD8^+^ cells. The percentages of TCRβ^+^CD4^+^Foxp3‐GFP^‐^ CD44^hi^CD62L^lo^ and TCRβ^+^CD8^+^ CD44^hi^CD62L^lo^ cells (A) or Ki‐67^+^CD44^hi^ (B) cells were determined each day. The following numbers of mice were used at each time point. Day 0 (*n* = 14, PBS‐treated control mice), Day 1 (*n* = 3), Day 2 (*n* = 3), Day 3 (*n* = 3), Day 4 (*n* = 3), Day 5 (*n* = 5), and Day 6 (*n* = 6).

We first confirmed the effectiveness of the Treg depletion protocol in Foxp3‐DTR mice. The gating strategy is shown in Figure S1. One injection of diphtheria toxin (DT) resulted in almost complete depletion of Foxp3^+^ Treg cells, which was maintained for 6 days by intraperitoneal injections every other day (Figure S2A,B). We measured the activation of CD4^+^Foxp3^−^ and CD8^+^ Tconv by induction of expression of high levels of CD44 (CD44^hi^) and by incorporation of Ki‐67 which detects recently divided cells. Increases of both parameters on both T‐cell subsets could be observed as early as day 3 after Treg depletion and peaked between days 4 and 6 (Figure 1A,B).

We also evaluated the cytokine‐producing capacity of the activated T‐cell subsets on day 6 after Treg depletion. CD4^+^Foxp3^−^CD44^hi^ T cells produced a slightly higher level of IFN‐γ and a comparable level of TNFα but these differences were not statistically significant, while the production of these cytokines by CD8^+^CD44^hi^ T cells from the Treg‐depleted mice was more pronounced (Figure S2C). High levels of IFN‐γ could be detected in plasma and the levels of IFN‐γ‐dependent chemokines CXCL9 and CXCL10 were also increased but the increases were not statistically significant (Figure S2D). Modest increases in plasma levels of TNFα and IL‐6 were present, but the plasma levels of IL‐2 were low (Figure S2D).

### 2.2 Role of APC in the Treg Depletion‐Induced Activation Cascade

**FIGURE 2:**
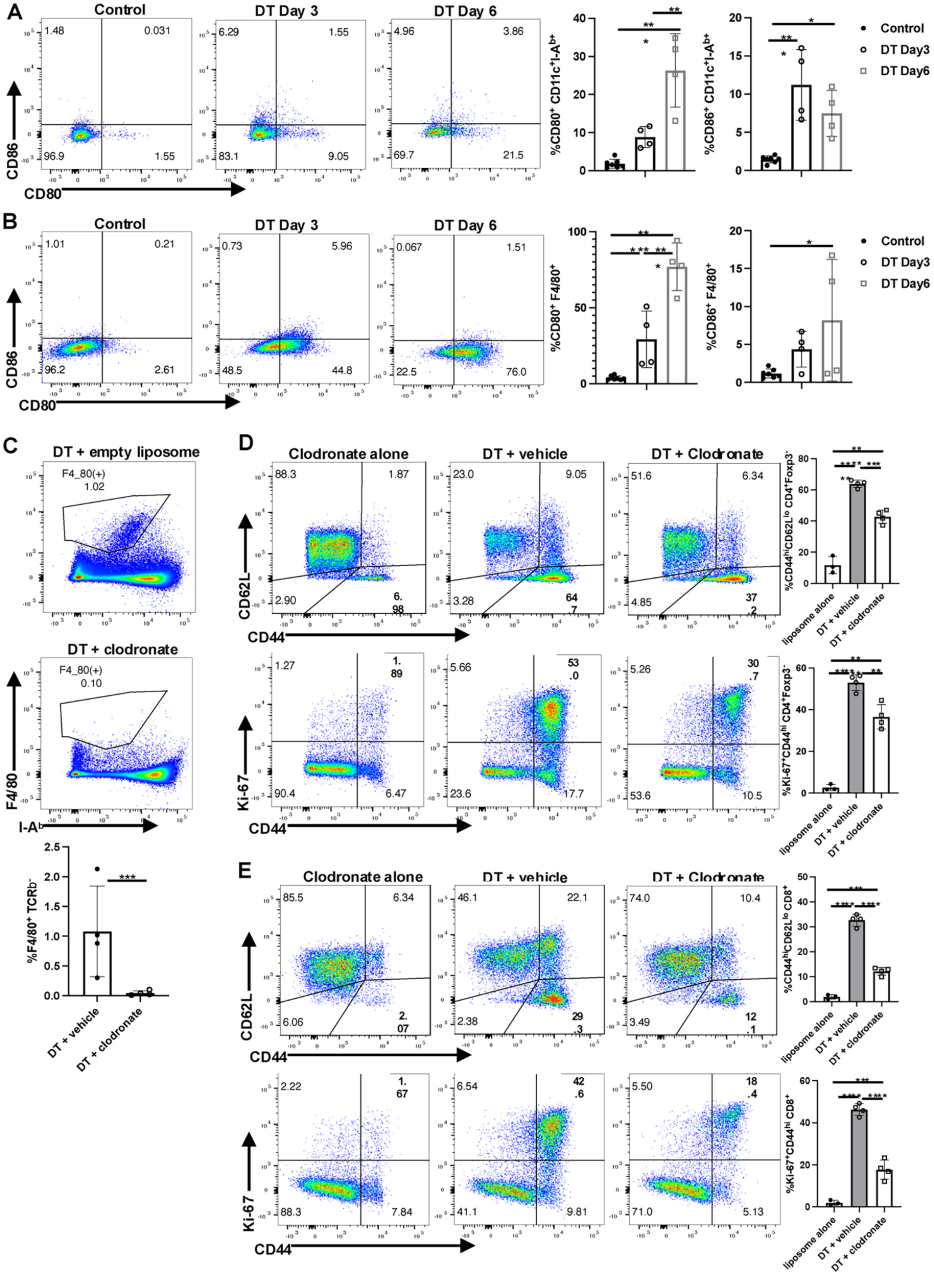
Role of APC in the T‐cell activation cascade after the Treg depletion. Expression of CD80 and CD86 were analyzed on CD11c^+^I‐A^b+^ (A) and F4/80^+^ (B) splenocytes from control or DT‐treated mice (day 3 or day 6 post‐DT). (C) Mice were treated with DT and empty liposomes or DT and clodronate liposomes. On day 5 after treatment, splenocytes were stained with F4/80. CD4^+^Foxp3^−^ (D) or CD8^+^ (E) splenocytes from recipients of empty liposomes or clodronate liposomes were analyzed on day 5 after DT treatment for expression of CD44/CD62L and Ki‐67. Each symbol represents an individual mouse, and data are pooled from at least two independent experiments. *p < 0.05, **p < 0.01, ***p < 0.001, ****p < 0.0001.

As APCs are required to induce the activation of naïve T cells [13], we initially examined costimulatory molecule expression on both splenic DC and macrophages at early time points after Treg depletion (Figure 2A,B). The expression levels of CD80 and CD86 on DCs were both increased 3 days after Treg depletion. CD40 and PD‐L1 expression were upregulated on day 6 after Treg depletion (Figure S3A), while upregulated expression of MHC class I and II were observed earlier on day 3 (Figure S3B).

Treatment of wild‐type mice with DT alone did not result in upregulation of CD80/CD86 expression ruling out the possibility that DT alone may induce inflammation (Figure S3C). To rule out the possibility that upregulation of CD80/CD86 was secondary to cell death induced by DT treatment, we generated heterozygous female mice expressing one wild‐type Foxp3 allele and one Foxp3‐DTR allele. Treatment of these mice with DT did not result in activation of Tconv as expected and did not result in upregulation of CD80/CD86 (Figure S3D).

Splenic F4/80^+^ macrophages also showed increased expression of CD80/CD86 (Figure 2B) with a dramatic increase in expression of CD80 on day 3 and day 6 after Treg depletion. Macrophages also exhibited increased levels of MHC class II on day 5 (Figure S3E). The marked increase in expression of CD80/CD86 on F4/80^+^ cells raised the possibility that CD80/CD86^+^ macrophages were playing a critical role in Tconv cell activation. To address this possibility, we depleted macrophages using clodronate liposomes during Treg depletion. Clodronate treatment was quite effective at depleting F4/80^+^ cells (Figure 2C). Mice simultaneously treated with clodronate and DT exhibited approximately a one‐third reduction in activation of CD4^+^Foxp3^−^ T cells compared with mice treated with DT alone (Figure 2D). The effects of clodronate depletion of macrophages were much greater on the activation of CD8^+^ T cells as a greater than 50% reduction in CD8^+^ T‐cell activation was seen (Figure 2E). Taken together, these studies indicate that macrophages play a prominent role in inducing activation of Tconv following Treg depletion.

### 2.3 Role of T Cells and T‐Cell‐Derived Cytokines in the Treg Depletion‐Induced Activation Cascade

**FIGURE 3:**
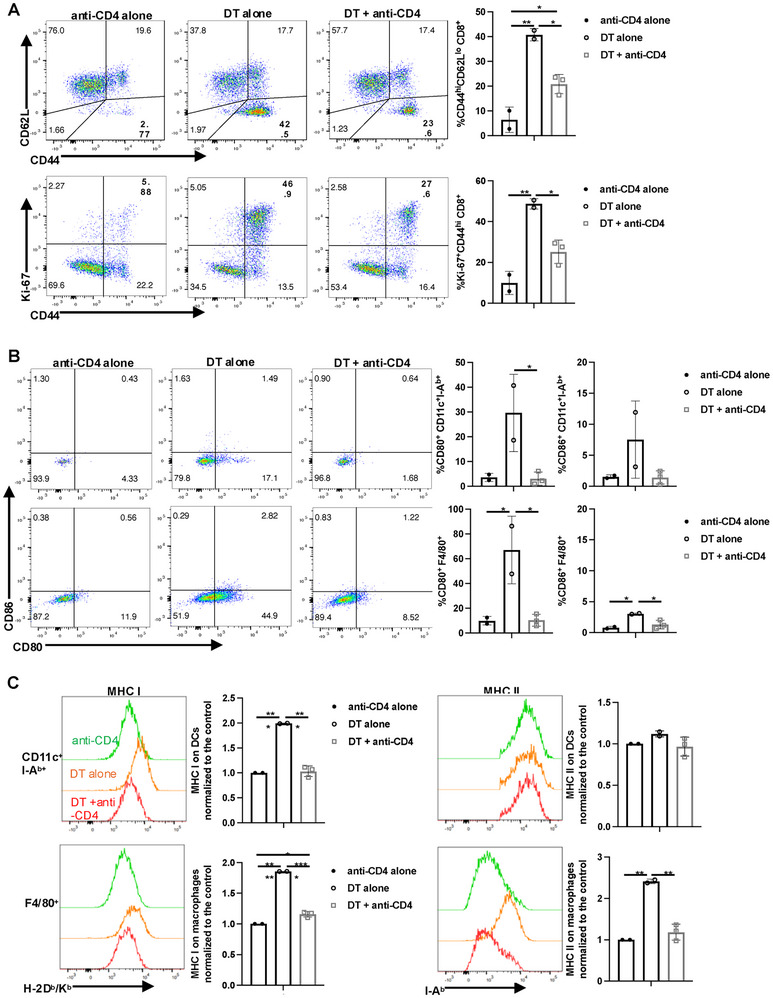
Role of CD4^+^ T cells in post‐DT immune activation. (A) Foxp3‐DTR mice were injected i.p. with DT with or without CD4‐depleting antibody (GK1.5, 500 µg) every other day and the expression of CD44/CD62L and Ki‐67 on CD8^+^ T cells were determined on day 5. (B) The expression of CD80/CD86 and (C) the expression of MHC‐I and MHC II on CD11c^+^ and F4/80^+^ cells were analyzed on day 5. Bar graphs compare MFI normalized to the control group. Each symbol represents an individual mouse, and data are pooled from two independent experiments. *p < 0.05, **p < 0.01, ***p < 0.001, ****p < 0.0001.

Even after several decades of studies, it remains unclear whether the suppressive function of Tregs in vivo acts directly on CD4^+^ or CD8^+^ T cells [10, 11]. To determine if TCR signaling was also induced by Treg depletion, we examined several early signaling events in both CD4^+^Foxp3^−^ and CD8^+^ T cells. Increased phosphorylation of ZAP‐70 (pZAP‐70) and increases of pAKT, p‐mTOR, and pS6 were observed on day 3 on both CD4^+^Foxp3^−^ and CD8^+^ T cells after Treg depletion (Figure S4A–D). The increases were slightly more prominent in CD4^+^Foxp3^−^ T cells compared with CD8^+^ T cells. This result raises the possibility that the activation of self‐reactive T cells may represent the very first step of the initiation of the Treg‐depletion immune cascade with subsequent activation of MHC class II^+^ APC functioning as a feed‐forward loop. Indeed, the Rudensky group commented that antibody‐mediated depletion of CD4^+^ T cells reversed the increased DC number after Treg depletion in the Foxp3‐DTR mouse [6]. To directly test this hypothesis, we used the anti‐CD4 depleting antibody (clone GK1.5) to deplete CD4^+^ T cells during DT‐mediated Treg depletion. Even though we could not achieve complete depletion of CD4^+^ T cells under these conditions (Figure S4E), activation of CD8^+^ T cells was decreased by half as measured by upregulation of CD44 expression and Ki‐67 incorporation (Figure 3A). Most importantly, the upregulation of CD80/CD86 expression (Figure 3B) and MHC I/MHC II expression on both DCs and macrophages was completely reversed (Figure 3C). These results imply a significant role for CD4^+^ T cells in the activation of APC and subsequent T‐cell activation.

**FIGURE 4:**
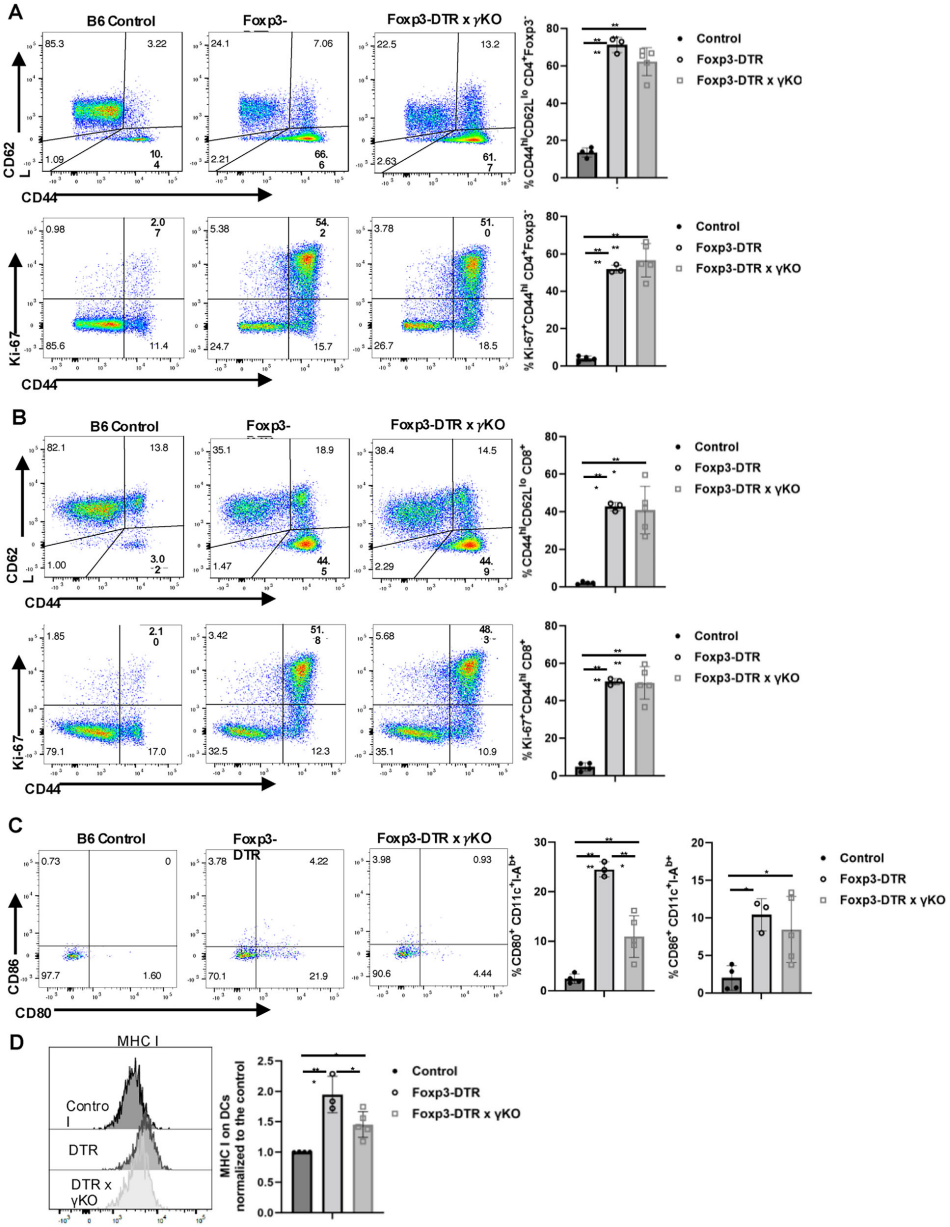
Analysis of the role of IFN‐γ using Foxp3‐DTR x IFN‐γ knock‐out mice. DT was administered to B6 control, Foxp3‐DTR, and Foxp3‐DTR x IFN‐γ Knock out (Foxp3‐DTR x γKO) mice. The expression of CD44/CD62L and Ki‐67 on CD4^+^Foxp3^−^ cells (A) and CD8^+^ T cells (B), the expression of CD80/CD86, and MHC class I on CD11c^+^ cells (C, D) were assayed on day 5 after DT treatment. (D) Bar graphs compare MFI normalized to the control group. Each symbol represents an individual mouse, and data are pooled from three independent experiments. *p < 0.05, **p < 0.01, ***p < 0.001, ****p < 0.0001.

As activated T cells can produce IFN‐γ which can induce activation of APCs to increase costimulatory CD80/CD86 expression [14, 15], and because serum IFN‐γ was markedly increased after Treg deletion (Figure S2D), we generated Foxp3‐DTR‐IFN‐γ^−/−^ mice. Surprisingly, the magnitude of T‐cell activation as measured by upregulation of CD44 or Ki‐67 incorporation on either CD4^+^Foxp3^−^ or CD8^+^ T cells was equivalent to that seen in wild‐type Foxp3‐DTR mice (Figure 4A,B). In contrast, the marked upregulation of CD80 expression observed on CD11c^+^ DCs post‐DT treatment was greatly reduced in cells derived from the Foxp3‐DTR‐IFN‐γ^−/−^ mice (Figure 4C). A modest reduction in the levels of MHC I was also observed (Figure 4D). Thus, T‐cell‐derived IFN‐γ likely plays a prominent role in potentiating the effects of Treg depletion on the augmentation of costimulatory molecule expression on APC but is not involved in the initial stages of T‐cell activation.

### 2.4 Enhanced Availability of IL‐2 Does Not Mediate T‐Cell Proliferation After Treg Depletion

**FIGURE 5:**
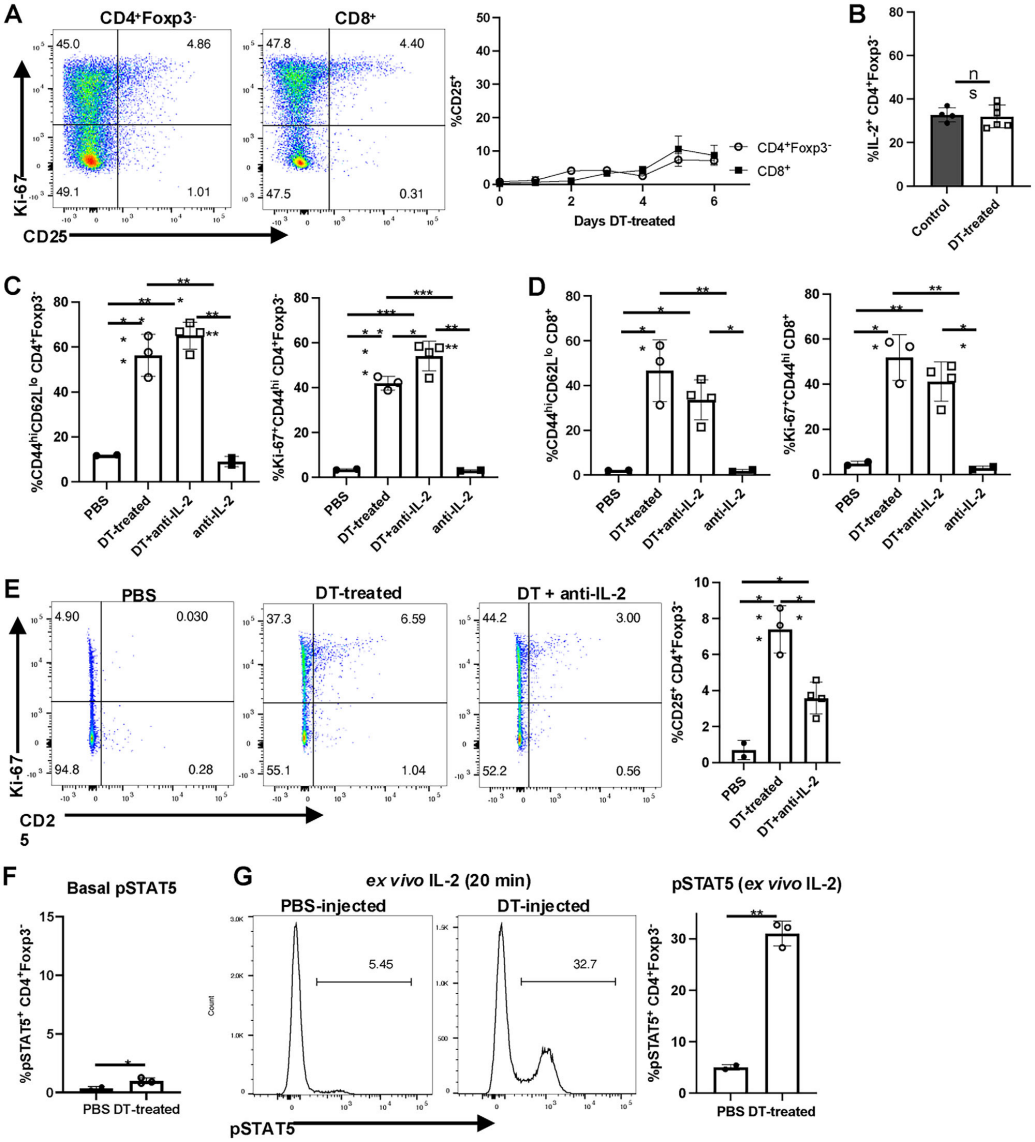
IL‐2 plays a minor role in T‐cell activation after DT treatment. (A) Expression of Ki‐67 and CD25 on CD4^+^Foxp3^−^ and CD8^+^ T cells after 6 days of DT treatment. Time course of expression of CD25 on CD4^+^Foxp3^−^ and CD8^+^ T cells after DT treatment. The representative 2D plot is from day 6 splenocytes gated on TCRβ^+^CD4^+^Foxp3^−^ cells or TCRβ^+^CD8^+^ cells. (B) IL‐2 production by CD4^+^ Foxp3^−^ T cells from control and DT‐treated mice on day 6 was measured by intracellular staining. (C–E) Foxp3‐DTR mice were i.p injected with DT with or without the anti‐IL‐2 neutralizing mAb (JES6, 200 µg) every other day. Expression of CD44/CD62L and Ki‐67 were analyzed on CD4^+^Foxp3^−^ (C) and CD8^+^ T cells (D) on day 6. (E) Expression of CD25 and Ki‐67 on CD4^+^ Foxp3^−^ T cells on day 6. (F‐G) Day 6 splenocytes were used to measure the phosphorylation of STAT5. (F) Expression of pSTAT5 on CD4^+^ Foxp3^−^ T cells from untreated or DT‐treated mice. (G) pSTAT5 expression in response to IL‐2 (50 U/mL) on CD4^+^ Foxp3^−^ T cells from control and DT‐treated mice. Each symbol represents an individual mouse, and data are pooled from at least two independent experiments. *p < 0.05, **p < 0.01, ***p < 0.001, ****p < 0.0001, ns = not significant.

Several studies have suggested that consumption of IL‐2 by Tregs is one of the major mechanisms by which Treg mediate their suppressive functions [16, 17, 18, 19]. Treg depletion might therefore result in enhanced availability of IL‐2 or even an “IL‐2 storm” resulting in the enhanced proliferation of both CD4^+^ and CD8^+^ T cells. As IL‐2 functions by upregulating IL‐2Rα (CD25) expression, it was surprising that only a low percentage of CD25^+^CD4^+^Foxp3^−^ or CD25^+^CD8^+^ T cells could be detected at any time point after Treg depletion (Figure 5A). Furthermore, IL‐2 production by CD4^+^Foxp3^−^ T cells was not increased (Figure 5B).

To further evaluate the contribution of IL‐2 in the T‐cell activation cascade provoked by Treg depletion, we examined the effect of anti‐IL‐2 blocking mAbs on the upregulation of CD44 expression and Ki‐67 incorporation. Treatment of Foxp3‐DTR mice with anti‐IL‐2 alone resulted in a decrease in Treg as described previously [20] indicating that the mAb is biologically active (Figure S3E). Simultaneous treatment with DT and anti‐IL‐2 mAbs had very little if any effect on the upregulation of CD44 expression or Ki‐67 incorporation induced by Treg depletion on either CD4^+^Foxp3^−^ (Figure 5C) or CD8^+^ (Figure 5D) T cells. The anti‐IL‐2 mAb did inhibit the modest induction of CD25 expression during DT treatment (Figure 5E). If IL‐2 was also playing a role in T‐cell activation following Treg depletion, one might predict that freshly explanted T cells from Treg‐depleted mice would express high levels of pSTAT5. However, CD4^+^Foxp3^−^ cells expressed only low levels of pSTAT5 (Figure 5F) but did exhibit enhanced responsiveness to exogenous IL‐2 (Figure 5G). Taken together, these studies demonstrate a minimal contribution of IL‐2 to T‐cell activation post‐Treg depletion.

### 2.5 Critical Contribution of Co‐Stimulation to Treg Depletion‐Mediated T‐Cell Activation

**FIGURE 6:**
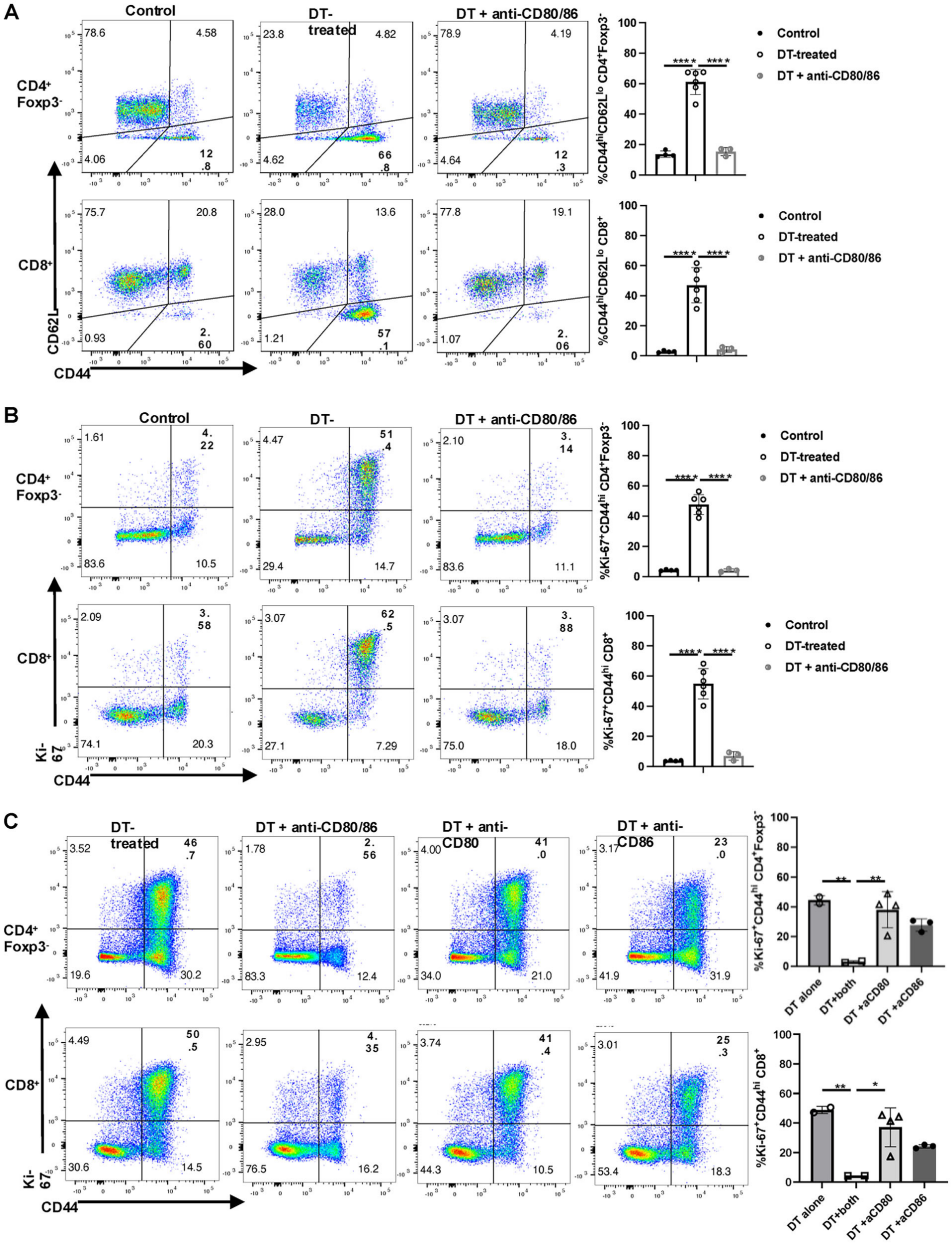
Critical role of CD80/CD86 on the induction of T‐cell activation. Foxp3‐DTR mice were injected i.p. with DT with or without anti‐CD80/CD86 mAbs (1G10 and GL‐1, 100 µg of each/mouse). Expression of CD44/CD62L (A) and Ki‐67 (B) on CD4^+^Foxp3^−^ and CD8^+^ T cells on day 6. (C) Foxp3‐DTR mice were injected i.p. with DT with or without the combination of CD80/CD86, anti‐CD80 (100 µg/mouse) alone or anti‐CD86 (100 µg/mouse) alone. Expression of CD44/CD62L and Ki‐67 were measured on CD4^+^Foxp3^−^ and CD8^+^ T cells on day 6. Each symbol represents an individual mouse, and data are pooled from at least two independent experiments. ****p < 0.0001.

The lack of evidence for IL‐2 as a causative factor in mediating the profound increase in T‐cell proliferation post‐Treg depletion raised the possibility that co‐stimulation alone or co‐stimulation in concert with TCR signaling was driving the response. To determine the role of CD80/CD86 mediated co‐stimulation, we depleted Treg cells and simultaneously treated the mice with blocking mAbs to CD80/CD86. All the parameters of immune cell activation induced by Treg depletion were completely reduced to the levels seen in control mice including upregulation of CD44 expression and enhanced Ki‐67 incorporation by CD4^+^Foxp3^−^ and CD8^+^ T cells (Figure 6A,B). The increases in pSTAT5 in response to exogenous IL‐2 in both CD4^+^Foxp3^−^ and CD8^+^ T cells were reduced to basal level (Figure S5A). In addition, enhanced cytokine production by CD44^hi^CD8^+^T cells and serum cytokine levels were normalized (Figure S5B,C). Although it is difficult to assay the effects of the anti‐CD80/CD86 mAbs on CD80/CD86 expression, the upregulated levels of MHC class I and II on APCs induced by Treg deletion were reversed, and the levels of CD40 and PD‐L1 expression normalized (Figure S5D,E). Unlike the complete reversal of Tconv activation by anti‐CD80/86, anti‐CD80 alone only slightly reduced T‐cell activation and anti‐CD86 moderately reduced the T‐cell activation provoked by the Treg depletion (Figure 6C). Taken together, CD80/CD86‐CD28 interactions appear to play a nonredundant role in the initiation of all aspects of immune system activation post‐Treg depletion.

### 2.6 A Limited Window Exists for Preventing Immune Activation by Blocking Co‐Stimulation

**FIGURE 7:**
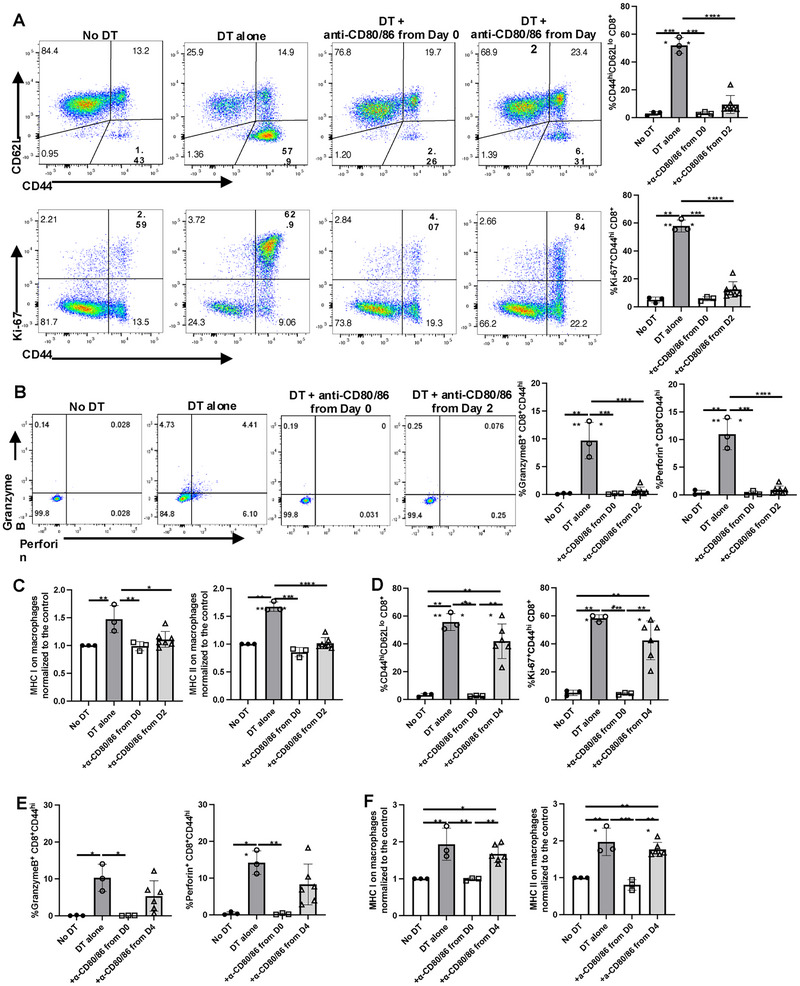
A limited window exists for reversal of cellular activation by anti‐CD80/CD86. Foxp3‐DTR mice were injected i.p. with DT starting on day 0 with or without anti‐CD80/CD86 from day 0 or day 2. (A) Expression of CD44/CD62L and Ki‐67 and (B) Granzyme B and perforin production by CD8^+^CD44^hi^ T cells were measured on day 5. (C) Expression of MHC I and MHC II on F4/80^+^ cells on day 5. (D–F) Foxp3‐DTR mice were injected i.p. with DT starting on day 0 with or without anti‐CD80/CD86 from day 0 or day 4. Expression of CD44/CD62L, Ki‐67 on CD8^+^ T cells (D), granzyme B and perforin on CD8^+^CD44^hi^ cells (E), and MHC I/II on F4/80^+^ cells (F) were measured on day 7. Each symbol represents an individual mouse, and data are pooled from three independent experiments. *p < 0.05, **p < 0.01, ***p < 0.001, ****p < 0.0001.

The remarkable therapeutic efficacy of anti‐CD80/CD86 mAbs in preventing immune activation post‐Treg depletion raised the possibility that blockade of the delivery of co‐stimulatory signals may be used to treat ongoing autoimmunity secondary to complete deletion of Treg or dysfunctional Treg. We delayed treatment with anti‐CD80/CD86 for 48 h after the initial administration of DT. When we assayed the mice on day 5 (3 days after the start of the delayed blockade), the increases in CD44 expression and Ki‐67 incorporation in CD8^+^ T cells (Figure 7A) and CD4^+^Foxp3^−^ cells (data not shown) were almost reduced to basal levels in the treated mice. Similarly, increased levels of granzyme B and perforin production by CD8^+^ T cells and MHC I/II expression on APC were reduced to basal levels (Figure 7B,C). As day 2 postdepletion is the time point when APC activation has just started and no changes in T‐cell activation are apparent (Figure 1A,B), we next delayed treatment with anti‐CD80/CD86 until day 4 postinitiation of DT. When checked 3 days after the start of the delayed blockade on day 7, only modest reductions in CD44 expression and Ki‐67 incorporation by CD8^+^ T cells were observed (Figure 7D). Granzyme B and perforin production by CD8^+^ T cells and MHC I/II levels on APC were only slightly decreased compared with animals treated with DT alone (Figure 7E,F). Thus, anti‐CD80/CD86 treatment is quite effective when given 48 h after the start of DT administration, but effectiveness is rapidly lost when anti‐CD80/CD86 treatment is delayed for 96 h after DT administration.

## 3 Discussion

Treg depletion represents a powerful method to induce immune activation involving multiple cell types ultimately leading to the death of the treated animal. We initially focused on the earliest events in the activation cascade which we could detect by phenotypic study of both T cells and APC populations to determine the mechanism of suppression utilized by Treg cells in mediating immune homeostasis in the steady state. The most striking finding in our studies was that all aspects of T‐cell and APC activation including CD44 and Ki‐67 expression on T cells, enhanced Zap70 phosphorylation on T cells, as well as all signs of APC activation including upregulation of MHC I and MHC II expression on APC were blocked by treatment with anti‐CD80/CD86.

Treg cells are the main cell type expressing CTLA‐4 in the steady state and it was proposed more than twenty years ago that Tregs suppress Tconv cells by outcompeting CD28 for the binding to CD80/CD86 on APC [4, 21]. In addition to the occupancy of CD80/CD86, CTLA‐4 functions extrinsically by capturing CD80/CD86 from APC by a process of transendocytosis ultimately leading to intracellular degradation of the captured CD80/CD86 molecules and depletion of APC‐delivered costimulatory signals [22, 23, 24, 25]. Taken together, our results support the view that CTLA‐4 expression on Tregs in the steady state plays a role in “tuning” the baseline level of CD80/CD86 expression and thereby prevents T‐cell activation by autoantigens. Rapid depletion of Treg eliminates this gate‐keeper role of Treg in controlling the levels of CD80/CD86 expression and thereby facilitating the activation of autoreactive T cells.

This initial step of activation of autoreactive cells by autoantigen and co‐stimulation is rapidly followed by a cascade of events augmented by the production of IFN‐γ by memory T cells and further upregulation of CD80/CD86 expression. Previous studies have raised the possibility that the abrogation of Treg suppression of IFN‐γ production by memory CD4^+^ or CD8^+^ T cells may be the initiating event in the activation cascade [6]. Antibody depletion of CD4^+^ T cells and clodronate depletion of macrophages resulted in very similar results: ∼50% decrease in the upregulation CD44 and Ki‐67 on CD8^+^ T cells (Figures 2E and 3A). The effects of CD4 depletion were more pronounced on the upregulation of CD80/CD86 on APCs (Figure 3B). When we bred the Foxp3‐DTR mice to IFN‐γ^−/−^ mice, T‐cell activation as measured by upregulation of CD44 expression and incorporation of Ki‐67 were completely independent of IFN‐γ, while upregulation of CD80/CD86 expression was IFN‐γ‐dependent (Figure 4). We conclude that T‐cell production of IFN‐γ likely plays a prominent role in upregulating costimulatory molecules, but memory T cell‐derived IFN‐γ is not the initiating event in the activation cascade.

Several studies [16‐19] have suggested that one of the major pathways by which Treg mediate their suppressor function both in vitro and in vivo is secondary to their high expression of CD25 which mediates their competition for IL‐2 produced by T effector cells. Complete depletion of Treg might therefore result in loss of competition and a rapid increase in systemic IL‐2 (an “IL‐2 storm”) which could potentially result in the activation of both CD4^+^ and CD8^+^ memory T cells. We found little evidence for such an activation cascade. In fact, one of the more striking findings is that only a low percentage of Ki‐67^+^ CD4^+^ or CD8^+^ T cells expressed CD25 (Figure 5A) indicating that IL‐2 was not mediating T‐cell proliferation post‐Treg depletion. Similar results on CD25 expression were reported in the original studies of Kim et al. [6]. Neutralization of IL‐2 did decrease the modest increase in CD25 expression (Figure 5E), but it did not affect the activation of Tconv. It is thus likely that T‐cell activation and proliferation post‐Treg depletion are cytokine‐independent and are driven by autoantigen‐driven TCR signaling alone, by co‐stimulatory signals in concert with TCR stimulation, or even by co‐stimulatory signals alone.

While our studies strongly support a critical role for CTLA‐4 in Treg function, the studies of Patterson et al. [26] demonstrate that conditional deletion of CTLA‐4 in Treg in the adult mouse does not lead to autoimmunity, does not impair Treg suppressor function and confers resistance to EAE, have questioned the nonredundant function of CTLA‐4 in Treg function in the adult mouse. While this report proposes that conditional deletion of CTLA‐4 in the adult mouse results in Treg expansion and enhanced expression of inhibitory pathways, including IL‐10 production, enhanced LAG‐3 and PD‐1 expression which may compensate for loss of CTLA‐4 expression, further studies are required to resolve the differences between the deletion studies and our blocking experiments with anti‐CD80/CD86. As IL‐10 can regulate the expression of CD80/CD86 [15], the depletion of IL‐10‐producing Treg cells might be involved in the upregulation of CD80/CD86 on APCs in addition to the downregulation of CTLA‐4 by Treg. In a recent report [27], CTLA‐4 is shown to be more efficient in transendocytosis when engaged with CD86 than CD80. Consistent with this report, when we treated animals with either anti‐CD80 or anti‐CD86 in conjunction with DT, anti‐CD80 only slightly reduced T‐cell activation, while anti‐CD86 produced a moderate reduction of T‐cell activation (Figure 6C). However, the magnitude of reversal of T‐cell activation by anti‐CD86 alone was always much less than that observed with the combination treatment.

The role of quantitative differences in CTLA‐4 expression in controlling the development of autoimmunity is stronger in humans than mice [28]. Heterozygous CTLA‐4 deficient mice are normal [29], but humans with CTLA‐4 haploinsufficiency develop a severe autoimmune phenotype [30, 31]. Similarly, mice with a deficiency of lipopolysaccharide‐responsive beige‐like anchor (LRBA), a molecule that mediates endosomal recycling of CTLA‐4 to the cell surface are normal [32, 33], while humans with LRBA deficiency develop autoimmune manifestations [34, 35]. Whether the insufficient levels of CTLA‐4 in both conditions are secondary to reduced levels of CTLA‐4 on Treg or T effector cells or both remains unclear. We were unable to inhibit the T‐cell activation by delayed treatment with anti‐CD80/CD86 when Treg were completely depleted at 96 h. Nevertheless, the successful treatment of patients with partial defects [30, 31, 34, 35] in CTLA‐4 expression or autoimmune disease [36] with exogenous CTLA‐4‐Ig (Abatacept) is consistent with a model in which the combination of decreased levels of endogenous CTLA‐4 in combination with exogenous CTLA‐4 can restore immune homeostasis.

## 4 Data Limitations and Perspectives

The conclusions drawn in this study regarding the role of Treg in controlling CD80/CD86 expression and the role of co‐stimulation as a critical component of the T‐cell activation cascade only pertain to the immediate events seen post‐Treg depletion. The mechanisms involved in the long‐term role of Treg in controlling autoimmune diseases are likely considerably more complex as are the defects in Treg function seen in disease. Our attempts to perform long‐term studies of the effects of Treg depletion were not successful. Higher doses and prolonged administration of DT can result in the deaths of the recipients due to exuberant autoimmunity as described previously [6, 7]. In addition, in some of our attempts using prolonged administration of DT, Treg numbers returned and the animals also developed antibodies to DT. Although Treg depletion using Foxp3‐DTR mice is a valuable tool for short‐term studies, we believe that the goal of obtaining a mouse that lacks Treg and has a normal immune repertoire is impossible to achieve using this approach.

## 5 Materials and Methods

### 5.1 Mice

C57BL/6J, Foxp3‐DTR mice (B6.129(Cg)‐*Foxp3^3(DTR/GFP)Ayr^*/J), and Foxp3‐RFP(C57BL/6‐*Foxp3^1Flv^/J*) were purchased from Jackson Laboratory (stock # 016958 and 008374). IFN‐γ‐deficient mice were obtained from the NIAID contract facility maintained at Taconic. These mice were housed in the animal facility of the National Institutes of Allergy and Infectious Diseases under specific pathogen‐free conditions. Age‐ and sex‐matched female and male mice were used for each experiment. The mice used in this study were 6–17 weeks old. The animal protocol used in this study was approved by the Animal Care and Use Committee of the National Institute of Allergy and Infectious Diseases (LISB‐15E).

### 5.2 Treg Depletion and In Vivo Monoclonal Ab Treatment

Foxp3‐DTR mice were injected i.p. with diphtheria toxin (1 µg, List Biological Labs, #150) on day 0 and then 0.5 µg every other day. To neutralize cytokines or deplete specific kinds of cells in addition to Treg cells, recipient mice were injected with mAbs as indicated in the figure legends. The following mAbs were purchased from BioXcell; anti‐CD80 (clone 1G10), anti‐CD86 (clone GL‐1), anti‐IL‐2 (clone JES6), anti‐IFN‐𝛾 (clone XMG1.2), anti‐NK1.1 (clone PK136), anti‐CD8 (clone 2.43), and anti‐CD4 (clone GK1.5). Mice were injected i.p. with 100–500 µg of the mAbs in 100–200 µL of volume per injection. InVivoPure pH7.0 Dilution Buffer (BioXcell) was used for dilution of the antibodies and DT and for injection of the control mice. For depletion of macrophages, 200 µL of clodronate liposomes or control liposomes (Encapsula Nanosciences) were injected i.p.

### 5.3 Isolation of Splenic Leukocytes

Spleens from recipients were ground with a syringe plunger and filtered through a 70 µm strainer. Red blood cells were lysed by treatment with ACK lysing buffer (Gibco). Splenic DC and macrophages were isolated from mice that had been injected intrasplenically with PBS containing Liberase TL (40 µg/mL, Millipore Sigma) and DNAse I (200 µg/mL, Millipore Sigma) for 30 min at 37°C before cell isolation.

### 5.4 Cell Culture

Cells were cultured in a 5% CO_2_ at 37°C incubator in RPMI 1640 medium (Gibco) supplemented with 10 % fetal bovine serum, 10 mM HEPES, 1 mM sodium pyruvate, 2 mM of L‐glutamine, 1X Penicillin‐Streptomycin, 1X nonessential amino acids, and 50 µM of 2‐mercaptoethanol.

### 5.5 Flow Cytometry

Washed splenocytes were incubated with a Live/Dead fixable near‐IR dead cell staining kit (Thermo Fisher) for 20 min at room temperature (RT). After washing with FACS staining buffer (PBS containing 2% fetal bovine serum and 0.1% sodium azide), cells were treated with Fc blocking antibodies (2.4G2, BioLegend), and fluorochrome‐conjugated antibodies for 25 min at 4°C. For the intracellular staining of Ki‐67 and Foxp3, surface‐stained cells were fixed and permeabilized with eBioscience Foxp3/Transcription factor staining buffer set (Thermo Fisher) according to the manufacturer's instruction, then stained with antibodies at RT for 30 min. For the intracellular staining of cytokines, perforin, and granzyme B, splenocytes were incubated at 37°C overnight with an eBioscience cell stimulation cocktail plus protein transport inhibitors (Thermo Fisher). Harvested cells were stained with the Live/Dead kit and antibodies for surface markers, and fixed with BD Cytofix Fixation Buffer (BD Biosciences) at RT for 30 min, then stained for cytosolic molecules at RT for 30 min. For the staining of phosphorylated intracellular molecules, splenocytes were fixed with a prewarmed BD Cytofix Fixation Buffer at 37°C for 10 min. After washing with FACS staining buffer, cells were permeabilized with prechilled BD Perm Buffer III (BD Biosciences) at 4°C for 30 min. Then, washed cells were stained with fluorochrome‐conjugated antibodies at 4°C overnight. In the case of staining of phosphorylated STAT5, freshly explanted cells were incubated with 50 U/mL of recombinant human IL‐2 (provided by the National Cancer Institute) or complete media only for 20 min at 37°C before the fixation. Cells were acquired with BD FACSymphony (BD Biosciences) and analyzed using FlowJo software (Treestar) with adherence to the guidelines for the use of flow cytometry and cell sorting in immunological studies.

### 5.6 Antibodies Used for Flow Cytometry

The following anti‐mouse antibodies were purchased from BioLegend, BD biosciences, or Thermo Fisher. eF450‐anti‐CD4 (RM‐4‐5), APC‐anti‐CD4 (RM‐4‐5), PE‐Cy5‐anti‐CD4 (RM‐4‐5), BV650‐anti‐CD4 (RM‐4‐5), BUV737‐anti‐TCR (H57‐597), APC‐eF780‐anti‐TCR (H57‐597), PE‐Cy5‐CD8 (53‐6.7), PE‐CD8 (53‐6.7), PE‐Cy7‐CD8 (53‐6.7), PE‐anti‐CD25 (PC61.5), SB702‐anti‐CD25 (PC61.5), PE/Dazzle594‐anti‐B220 (RA3‐6B2), BB700‐anti‐CD44 (IM7), AF700‐anti‐CD44 (IM7), PE‐Cy7‐CD62L (MEL‐14), BV650‐anti‐NK1.1 (PK136), AF700‐anti‐Ki‐67 (SolA15), eF450‐anti‐CD11c (N418), APC‐anti‐F4/80 (BM8), BUV395‐anti‐CD11b (M1/70), FITC‐anti‐CD80 (16‐10A1), AF700‐anti‐CD86 (GL1), PE‐anti‐PD‐L1 (10F.9G2), PE‐Cy5‐anti‐CD40 (1C10), PE‐Cy7‐anti‐H‐2Kb/Db (28‐8‐6), BV650‐anti‐I‐Ab (25‐9‐17), eF450‐anti‐H‐2Db (28‐14‐8), PE‐Dazzle594‐anti‐I‐A/E (M5/114), FITC‐anti‐Foxp3 (FJK‐16S), APC‐anti‐pSTAT5 (SRBCZX), PE‐Cy7‐pZAP70 (17A/P‐ZAP70), PE‐pZAP70 (17A/P‐ZAP70), PE‐CF594‐anti‐pAKT (M89‐61), PE‐anti‐p‐mTOR (MRRBY), AF647‐anti‐p‐mTOR (O21‐404), PE‐Cy7‐anti‐pS6 (cupk43k), AF700‐anti‐TNF (MP6‐XT22), PE‐CF594‐anti‐IFN‐ (XMG1.2), PE‐anti‐IL‐2 (JES6‐5H4), PE‐Cy7‐anti‐GranzymeB (16G6), APC‐anti‐Perforin (S16009A).

### 5.7 Multiplex Cytokine Measurement

For the measurement of systemic cytokines and chemokines, the peripheral blood of control or DT‐treated mice was centrifuged, and plasma was used for the detection. BD Cytometric Bead Array Mouse Th1/Th2/Th17 kit (BD Biosciences) and customized LEGENDplex multiplex immunoassays kit (BioLegend) were used according to the manufacturer's instructions.

### 5.8 Statistical Analysis

Prism 9 (GraphPad) was used for analysis between groups. In general, an unpaired *t*‐test was done with Welch's correction to compare the two groups. Original one‐way ANOVA with Tukey's multiple comparison test was used to compare multiple groups with each other. *p < 0.05, **p < 0.01, ***p < 0.001, ****p < 0.0001, ns = not significant.

## Author Contributions

Yong‐Hee Kim and Ethan Shevach designed the experiments and wrote the manuscript. Yong‐Hee Kim and Abir Panda carried out the experiments.

## Ethics Statement

All of the animal studies reported in this paper were approved by the Animal Care and Use Committee of the National Institute of Allergy and Infectious Diseases.

## Conflicts of Interest

The authors declare no conflicts of interest.

### Peer Review

The peer review history for this article is available at https://publons.com/publon/10.1002/eji.202551771.

## Supporting information



Supplementary Materials

Supplementary Materials

Supplementary Materials

Supplementary Materials

Supplementary Materials

## Data Availability

The data that support the findings of this study are available from the corresponding author upon reasonable request.

## References

[1] S. Hori , T. Nomura , and S. Sakaguchi , “Control of Regulatory T Cell Development by the Transcription Factor Foxp3,” Science 299 (2003): 1057–1061. 12522256 10.1126/science.1079490

[2] J. D. Fontenot , M. A. Gavin , and A. Y. Rudensky , “Foxp3 programs the Development and Function of CD4+CD25+ Regulatory T Cells,” Nature Immunology 4 (2003): 330–336. 12612578 10.1038/ni904

[3] R. Khattri , T. Cox , S. A. Yasayko , and F. Ramsdell , “An Essential Role for Scurfin in CD4+CD25+ T Regulatory Cells,” Nature Immunology 4 (2003): 337–342. 12612581 10.1038/ni909

[4] T. Takahashi , T. Tagami , S. Yamazaki , et al., “Immunologic Self‐tolerance Maintained by CD25(+)CD4(+) Regulatory T Cells Constitutively Expressing Cytotoxic T Lymphocyte‐associated Antigen 4,” Journal of Experimental Medicine 192 (2000): 303–310. 10899917 10.1084/jem.192.2.303 PMC2193248

[5] R. S. Wildin , F. Ramsdell , J. Peake , et al., “X‐linked Neonatal Diabetes Mellitus, Enteropathy and Endocrinopathy Syndrome Is the Human Equivalent of Mouse scurfy,” Nature Genetics 27 (2001): 18–20. 11137992 10.1038/83707

[6] J. M. Kim , J. P. Rasmussen , and A. Y. Rudensky , “Regulatory T Cells Prevent Catastrophic Autoimmunity throughout the Lifespan of Mice,” Nature Immunology 8 (2007): 191–197. 17136045 10.1038/ni1428

[7] K. Lahl , C. Lodenkemper , C. Drouin , et al., “Selective Depletion of Foxp3^+^ Regulatory T Cells Induces a Scurfy‐Like Disease,” Journal of Experimental Medicine 204 (2007): 57–63. 17200412 10.1084/jem.20061852 PMC2118432

[8] M. Feuerer , Y. Shen , D. R. Littman , C. benoiost , and D. Mathis , “How Punctual Ablation of Regulatory T Cells Unleashes an Autoimmune Lesion Within the Pancreatic Islets,” Immunity 31 (2009): 654–664. 19818653 10.1016/j.immuni.2009.08.023 PMC2998796

[9] T. Chinen , P. Y. Volchkov , A. V. Chervonsky , and A. Y. Rudendsky , “A Critical Role for Regulatory T Cell‐mediated Control of Inflammation in the Absence of Commensal Microbiota,” Journal of Experimental Medicine 207 (2007): 2323–2330. 10.1084/jem.20101235 PMC2964571 20921284

[10] E. M. Shevach , “Mechanisms of Foxp3+ T Regulatory Cell‐Mediated Suppression,” Immunity 30 (2009): 636–645. 19464986 10.1016/j.immuni.2009.04.010

[11] D. A. A. Vignali , L. W. Collison , and C. J. Workman , “How Regulatory T Cells Work,” Nature Reviews Immunology 8 (2008): 523–532. 10.1038/nri2343 PMC2665249 18566595

[12] E. M. Shevach , R. S. McHugh , C. A. Piccirillo , and A. M. Thornton , “Control of T‐Cell Activation by CD4+CD25+ Suppressor T Cells,” Immunological Reviews 182 (2001): 58–67. 11722623 10.1034/j.1600-065x.2001.1820104.x

[13] M. Croft , D. D. Duncan , and S. L. Swain , “Response of Naive Antigen‐Specific CD4+ T Cells in Vitro: Characteristics and Antigen‐Presenting Cell Requirements,” Journal of Experimental Medicine 176 (1992): 1431–1437. 1357074 10.1084/jem.176.5.1431 PMC2119425

[14] A. S. Freedman , G. J. Freeman , K. Rhynhart , and L. M. Nadler , “Selective Induction of B7/BB‐1 on Interferon‐gamma Stimulated Monocytes: A Potential Mechanism for Amplification of T Cell Activation Through the CD28 Pathway,” Cellular Immunology 137 (1991): 429–437. 1716521 10.1016/0008-8749(91)90091-o

[15] L. Ding , P. S. Linsley , L. Y. Huang , R. N. Germain , and E. M. Shevach , “IL‐10 Inhibits Macrophage Costimulatory Activity by Selectively Inhibiting the Up‐Regulation of B7 Expression,” Journal of Immunology 151 (1993): 1224–1234. 7687627

[16] M. de la Rosa , S. Rutz , H. Dorninger , and A. Scheffold , “Interleukin‐2 Is Essential for CD4+CD25+ Regulatory T Cell Function,” European Journal of Immunology 34 (2004): 2480–2488. 15307180 10.1002/eji.200425274

[17] P. Pandiyan , L. Zheng , S. Ishihara , J. Reed , and M. J. Lenardo , “CD4+CD25+Foxp3+ Regulatory T Cells Induce Cytokine Deprivation‐Mediated Apoptosis of Effector CD4+ T Cells,” Nature Immunology 8 (2007): 1353–1362. 17982458 10.1038/ni1536

[18] Z. M. Y. G. Liu , N. Van Panhuys , A. G. Levine , A. Y. Rudensky , and R. N. Germain , “Immune Homeostasis Enforced by Co‐Localized Effector and Regualtory T Cells,” Nature 528 (2015): 225–230. 26605524 10.1038/nature16169 PMC4702500

[19] H. S. K. P. Wong , A. Gola , A. P. Baptista , et al., “A Local Regulatory T Cell Feedback Circuits Maintains Immune Homeostasis by Pruning Self‐activated T Cells,” Cell 184 (2021): 3981–3997. 34157301 10.1016/j.cell.2021.05.028 PMC8390950

[20] R. Setoguchi , S. Hori , T. Takahashi , and S. Sakaguchi , “Homeostatic Maintenance of Natural Foxp3(+) CD25(+) CD4(+) Regulatory T Cells by Interleukin (IL)‐2 and Induction of Autoimmune Disease by IL‐2 Neutralization,” Journal of Experimental Medicine 201 (2005): 723–735. 15753206 10.1084/jem.20041982 PMC2212841

[21] S. Read , V. Malmstrom , and F. Powrie , “Cytotoxic T Lymphocyte‐associated Antigen 4 Plays an Essential Role in the Function of CD25(+)CD4(+) Regulatory Cells That Control Intestinal Inflammation,” Journal of Experimental Medicine 192 (2000): 295–302. 10899916 10.1084/jem.192.2.295 PMC2193261

[22] K. Wing , Y. Onishi , P. Prieto‐Martin , et al., “CTLA‐4 Control Over Foxp3+ Regulatory T Cell Function,” Science 322 (2008): 271–275. 18845758 10.1126/science.1160062

[23] Y. Onishi , Z. Fehervari , T. Yamaguchi , and S. Sakaguchi , “Foxp3+ natural Regulatory T Cells Preferentially Form Aggregates on Dendritic Cells in Vitro and Actively Inhibit Their Maturation,” PNAS 105 (2008): 10113–10118. 18635688 10.1073/pnas.0711106105 PMC2481354

[24] O. S. Qureshi , Y. Zheng , K. Nakamura , et al., “Trans‐endocytosis of CD80 and CD86: A Molecular Basis for the Cell‐extrinsic Function of CTLA‐4,” Science 332 (2011): 600–603. 21474713 10.1126/science.1202947 PMC3198051

[25] M. Telguc , J. B. Wing , M. Osaki , J. Long , and S. Sakaguchi , “Treg‐expressed CTLA‐4depletes CD80/CD86 by Trogocytosis, Releasing Free PD‐L1 on Antigen‐Presenting Cells,” ProcNatl Sci USA 118 (2012): e2023739118. 10.1073/pnas.2023739118 PMC8325248 34301886

[26] A. M. Patterson , S. B. Lovitch , P. T. Sage , et al., “Deletion of CTLA‐4 on Regulatory T Cells During Adulthood Leads to Resistance to Autoimmunity,” Journal of Experimental Medicine 212 (2015): 1603–1621. 26371185 10.1084/jem.20141030 PMC4577848

[27] A. Kennedy , E. Waters , B. Roshanravan , et al., “Differences in CD80 and CD86 Transendocytosis Reveal CD86 as a Key Target for CTLA‐4 Immune Regulation,” Nature Immunology 23 (2022): 1365–1378. 35999394 10.1038/s41590-022-01289-w PMC9477731

[28] M. B. Jordan , “Modeling Primary Immune Regulatory Disorders: Ferrari or Yugo?,” Immunol and Cell Biol 95 (2017): 739–740. 28925382 10.1038/icb.2017.65

[29] P. Waterhouse , J. M. Penninger , E. Timms , et al., “Lymphoproliferative Disorders With Early Lethality in Mice Deficient in Ctla‐4,” Science 270 (1995): 985–988. 7481803 10.1126/science.270.5238.985

[30] H. S. Kuehn , W. M. Ouyang , B. Lo , et al., “Immune Dysregulation in human Subjects With Heterozygous Germline Mutations in CTLA4,” Science 345 (2014): 1623–1627. 25213377 10.1126/science.1255904 PMC4371526

[31] D. Schubert , C. Bode , R. Kenefeck , et al., “Autosomal Dominant Immune Dysregulation Syndrome in Humans With CTLA4 Mutations,” U Baumann 20 (2014): 1410–1416. 10.1038/nm.3746 PMC4668597 25329329

[32] D. L. Burnett , I. A. Parish , E. Masle‐Farquhar , R. Brink , and C. C. Goodnow , “Murine LRBA Deficiency Causes CTLA‐4 Deficiency in Tregs Without Progression to Immune Regulation,” Immunol and Cell Biol 95 (2017): 775–788. 28611475 10.1038/icb.2017.50 PMC5636941

[33] L. Gamez‐Diaz , J. Neumann , F. Jager , et al., “Immunological Phenotype of the Murine LRBA Knockout,” Immunol and Cell Biol 95 (2017): 789–802. 28652580 10.1038/icb.2017.52

[34] L. Gamez‐Diaz , D. August , P. Stepensky , et al., “The Extended Phenotype of LPS‐responsive Beige‐Like Anchor Protein (LRBA) Deficiency,” Journal of Allergy and Clinical Immunology 137 (2016): 223–230. 26768763 10.1016/j.jaci.2015.09.025

[35] G. Lopez‐Herrera , Q. P.‐H. G. Tampella , P. Herholz , et al., “Deleterious Mutations in LRBA Are Associated with a Syndrome of Immune Deficiency and Autoimmunity,” Am J HumGenet 90 (2012): 986–1001. 22608502 10.1016/j.ajhg.2012.04.015 PMC3370280

[36] M. Bonelli , L. Göschi , S. Blüml , et al., “Abatacept (CTLA‐4Ig) Treatment Reduces Cell Apoptosis and Regulatory T Cell Suppression in Patients with Rheumatoid Arthritis,” Rheumatology 55 (2016): 710–720. 26672908 10.1093/rheumatology/kev403

