# Supplementary figures and images for: Treg Control of CD80/CD86 Expression Mediates Immune System Homeostasis
Source: Eur J Immunol. 2025 May 9;55(5):e202551771. doi: 10.1002/eji.202551771 (PMC12064877; doi:10.1002/eji.202551771)

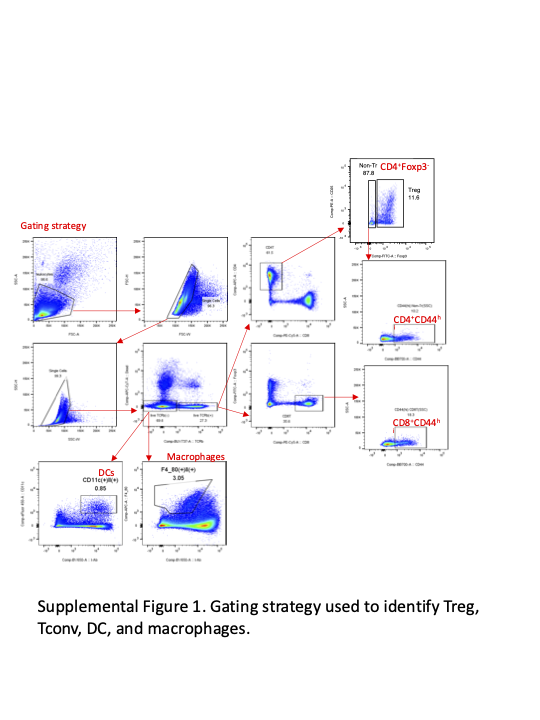

Supplement: Supplementary file 1 — Supplementary Materials [file EJI-55-e202551771-s005.tif]

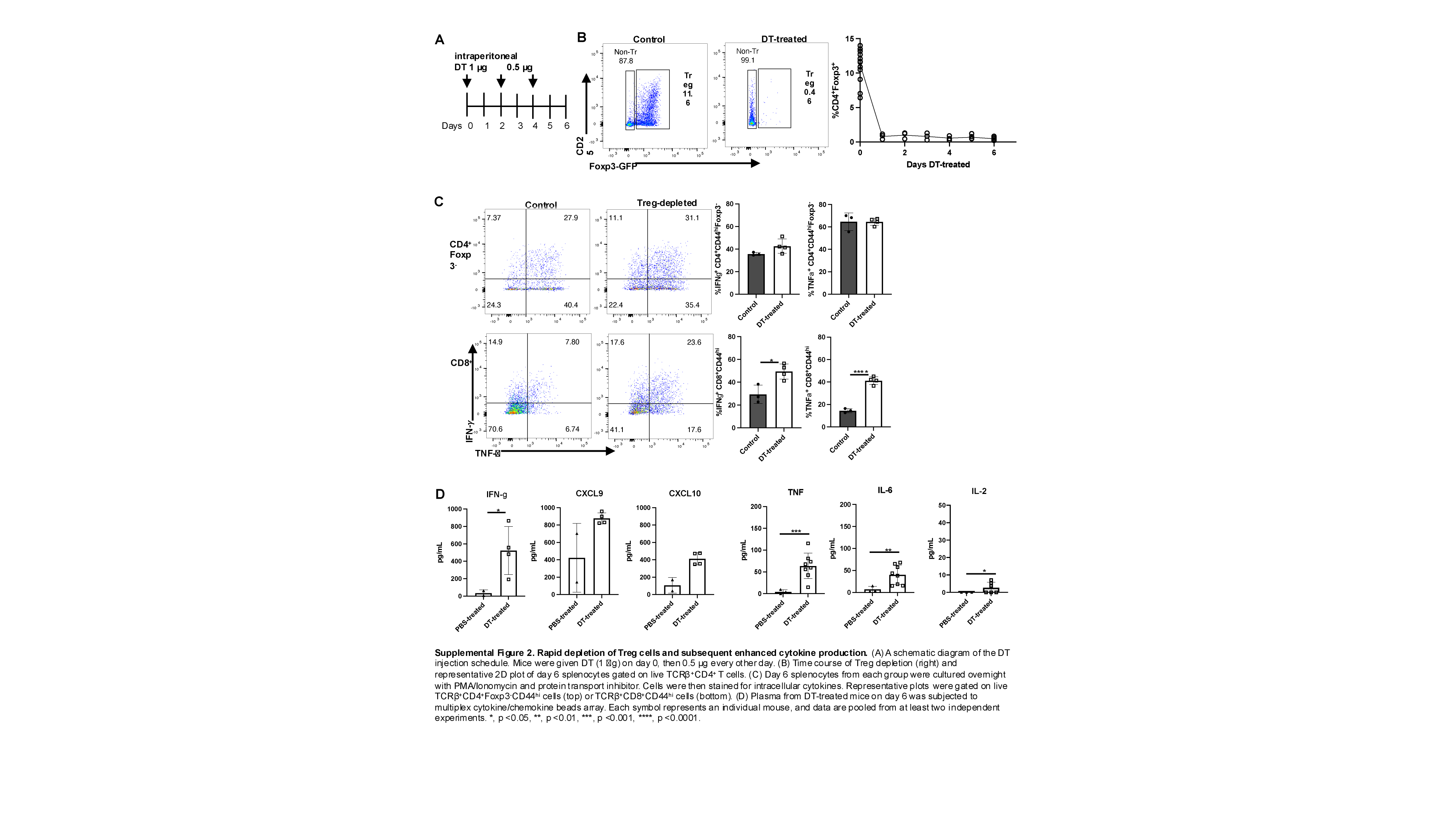

Supplement: Supplementary file 2 — Supplementary Materials [file EJI-55-e202551771-s003.tif]

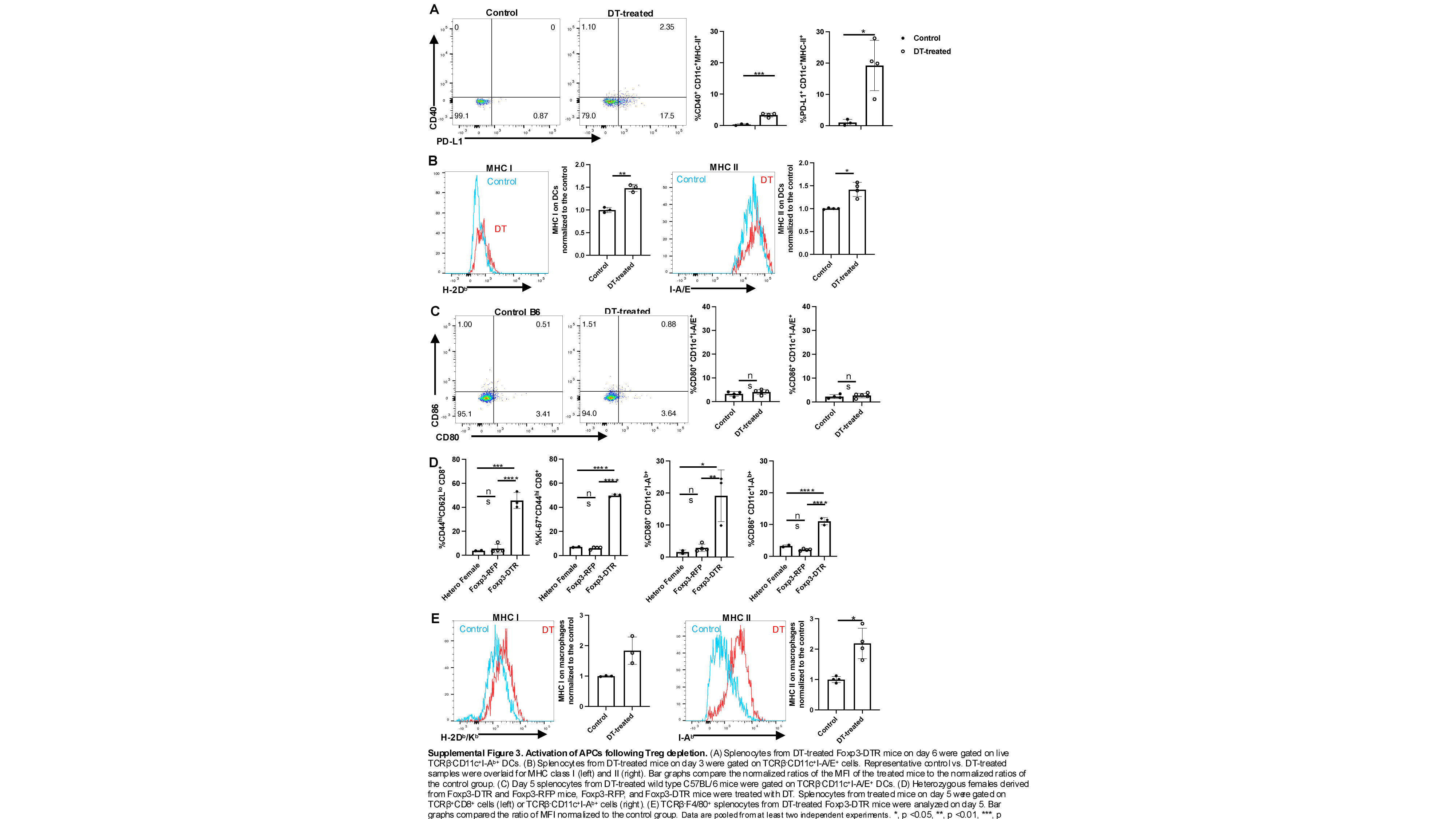

Supplement: Supplementary file 3 — Supplementary Materials [file EJI-55-e202551771-s002.tif]

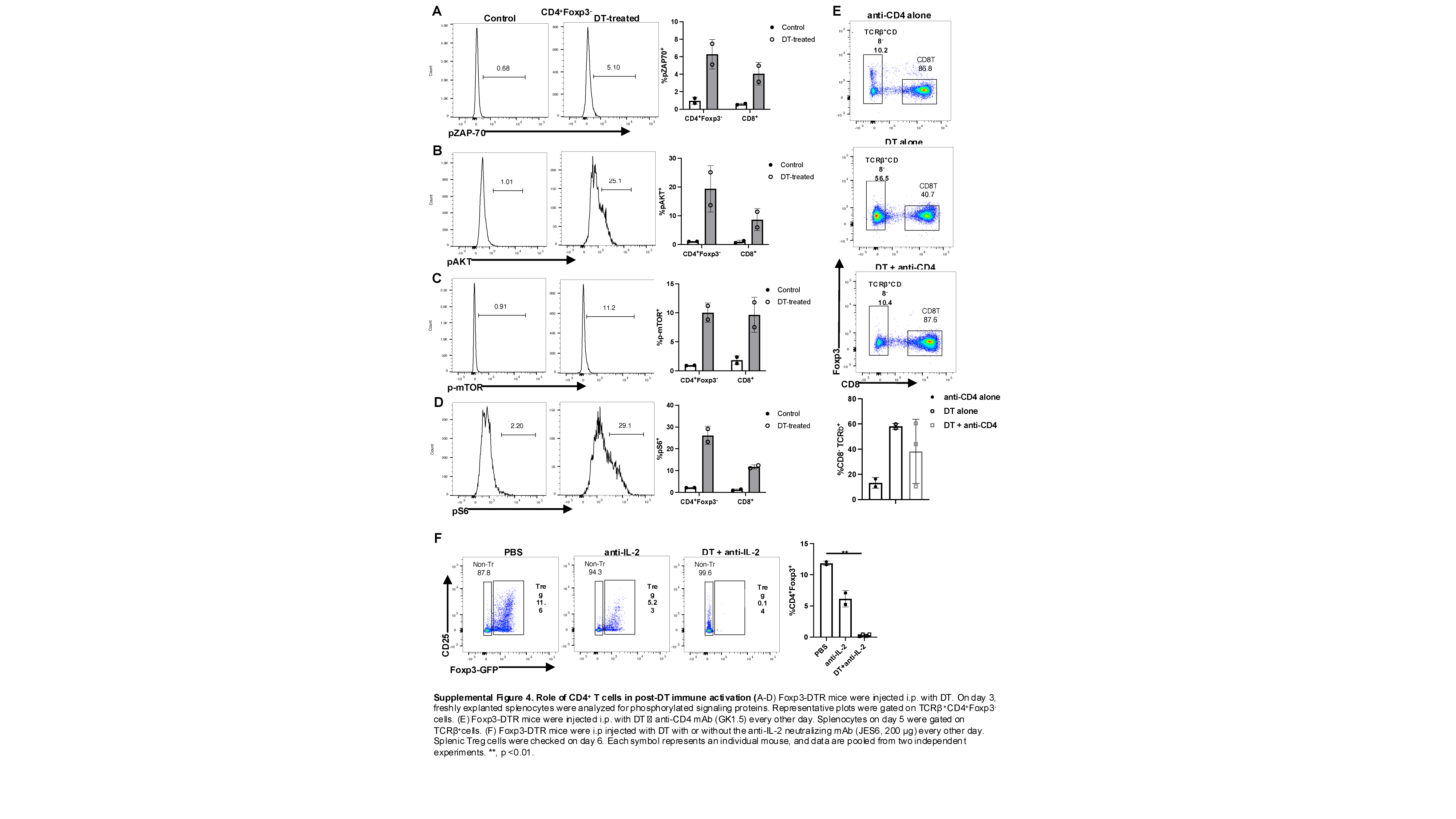

Supplement: Supplementary file 4 — Supplementary Materials [file EJI-55-e202551771-s001.tif]

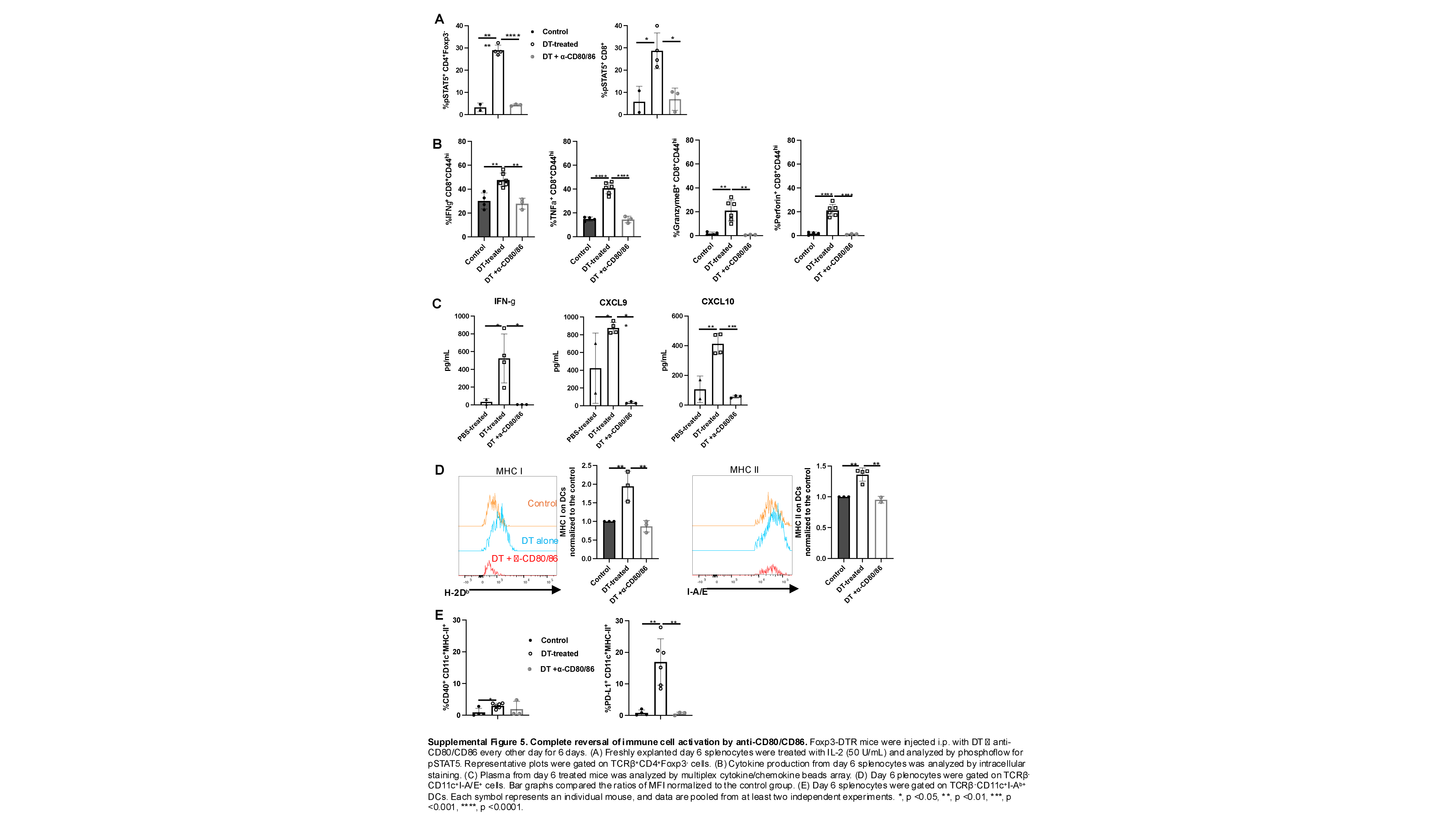

Supplement: Supplementary file 5 — Supplementary Materials [file EJI-55-e202551771-s004.tif]
